# 1-(2-Naphth­yl)-3-phenyl-3-(4,5,6,7-tetra­hydro-1,2,3-benzoselenadiazol-4-yl)propan-1-one

**DOI:** 10.1107/S1600536811027103

**Published:** 2011-07-13

**Authors:** J. Muthukumaran, M. Nachiappan, S. Chitra, P. Manisankar, Suman Bhattacharya, S. Muthusubramanian, R. Krishna, J. Jeyakanthan

**Affiliations:** a Centre for Bioinformatics, School of Life Sciences, Pondicherry University, Puducherry 605 014, India; b Department of Bioinformatics, Alagappa University, Karaikudi 630 003, India; c Department of Industrial Chemistry, Alagappa University, Karaikudi 630 003, India; d Department of Chemistry, Pondicherry University, Puducherry 605 014, India; e Department of Organic Chemistry, Madurai Kamaraj University, Madurai 625 021, India

## Abstract

In the title compound, C_25_H_22_N_2_OSe, the fused six-membered cyclo­hexene ring of the 4,5,6,7-tetra­hydro-1,2,3-benzoselenadiazole group adopts a near half-chair conformation and the five-membered 1,2,3-selenadiazole ring is essentially planar (r.m.s. deviation = 0.004 Å). There are weak inter­molecular C—H⋯O and C—H⋯π inter­actions in the crystal structure. Inter­molecular π–π stacking is also observed between the naphthyl units, with a centroid–centroid distance of 3.529 (15) Å.

## Related literature

For the biological importance of 1,2,3-selenadiazole derivatives, see: Kuroda *et al.* (2001 ▶); El-Bahaie *et al.* (1990 ▶); El-Kashef *et al.* (1986 ▶); Plano *et al.* (2010 ▶). For ring puckering analysis, see: Cremer & Pople (1975 ▶). For synthetic procedures, see: Al Arab (1989 ▶); Li *et al.* (2003 ▶); Qian *et al.* (2008 ▶); Xu *et al.* (2009 ▶).
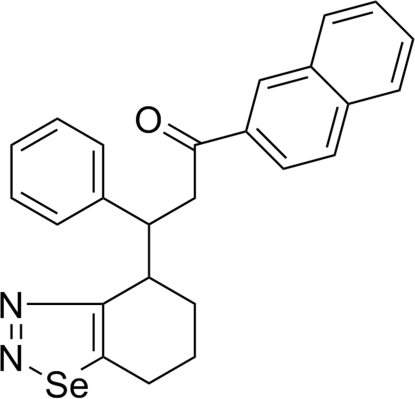


## Experimental

### 

#### Crystal data

C_25_H_22_N_2_OSe
*M* *_r_* = 445.41
Triclinic, 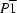

*a* = 8.0600 (4) Å
*b* = 10.1215 (5) Å
*c* = 13.0827 (6) Å
α = 81.479 (4)°
β = 76.478 (4)°
γ = 82.087 (4)°
*V* = 1020.33 (9) Å^3^
*Z* = 2
Mo *K*α radiation
μ = 1.86 mm^−1^
*T* = 293 K
0.4 × 0.3 × 0.2 mm

#### Data collection

Oxford Diffraction Xcalibur E diffractometer
Absorption correction: multi-scan (*CrysAlis PRO*; Oxford Diffraction, 2009 ▶) *T*_min_ = 0.658, *T*_max_ = 1.000
6762 measured reflections
3594 independent reflections
2770 reflections with *I* > 2σ(*I*)
*R*_int_ = 0.025

#### Refinement

*R*[*F* ^2^ > 2σ(*F* ^2^)] = 0.038
*wR*(*F* ^2^) = 0.096
*S* = 1.01
3594 reflections
262 parameters
H-atom parameters constrained
Δρ_max_ = 0.29 e Å^−3^
Δρ_min_ = −0.41 e Å^−3^

### 

Data collection: *CrysAlis PRO* (Oxford Diffraction, 2009 ▶); cell refinement: *CrysAlis PRO*; data reduction: *CrysAlis PRO*; program(s) used to solve structure: *SHELXS97* (Sheldrick, 2008 ▶); program(s) used to refine structure: *SHELXL97* (Sheldrick, 2008 ▶); molecular graphics: *ORTEP-3 for Windows* (Farrugia, 1997 ▶) and *PLATON* (Spek, 2009 ▶); software used to prepare material for publication: *PLATON*.

## Supplementary Material

Crystal structure: contains datablock(s) I, global. DOI: 10.1107/S1600536811027103/gk2375sup1.cif

Structure factors: contains datablock(s) I. DOI: 10.1107/S1600536811027103/gk2375Isup2.hkl

Supplementary material file. DOI: 10.1107/S1600536811027103/gk2375Isup3.cml

Additional supplementary materials:  crystallographic information; 3D view; checkCIF report

## Figures and Tables

**Table 1:** Hydrogen-bond geometry (Å, °) *Cg* is the centroid of the C16–C21 ring.

*D*—H⋯*A*	*D*—H	H⋯*A*	*D*⋯*A*	*D*—H⋯*A*
C20—H20⋯O1^i^	0.93	2.56	3.381 (3)	147
C22—H22⋯O1^i^	0.93	2.58	3.392 (4)	146
C4—H4*A*⋯*Cg*^ii^	0.97	2.63	3.584 (3)	167

Symmetry codes: (i) 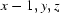; (ii) 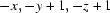.

**Fig. 1.**
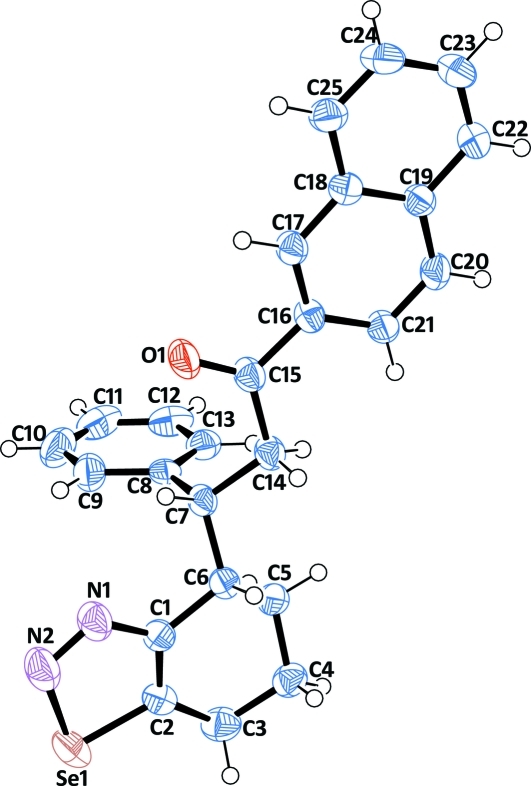
Molecular structure of the title compound, showing displacement ellipsoids drawn at the 50% probability level.

**Fig. 2.**
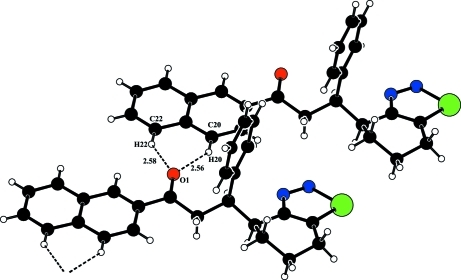
A weak intermolecular C—H···O interaction in title compound.

**Fig. 3.**
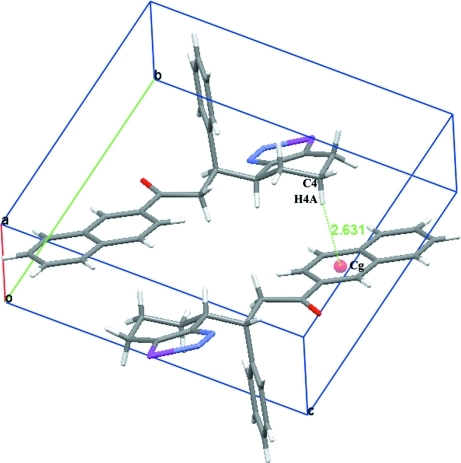
A weak intermolecular C—H···π interaction in title compound. Cg is the centroid of C16—C21 ring.

## supplementary crystallographic information

### Comment

Selenium containing heterocyclic compounds are of interest due to their biological applications. They posses various beneficial activities such as anti-fungal (Kuroda *et al.*, 2001), anti-bacterial (El-Kashef *et al.*, 1986), anti-microbial (El-Bahaie *et al.*, 1990), and anti-cancer (Plano *et al.*, 2010). Considering the importances of 1,2,3-selenadiazole derivatives, we report the structure of the title compound (Fig. 1). The five-membered 1,2,3-selenadiazole moiety (C1/N1/N2/Se1/C2) of the title compound adopts a planar conformation (r.m.s. deviation = 0.004 Å). Cremer & Pople puckering analysis (Cremer & Pople, 1975) cannot be performed to this moiety, for its weighted average absolute torsion angle is 0.62°, which is less than 5.0°. However, the fused six-membered ring (C1/C2/C3/C4/C5/C6) of 4,5,6,7-tetrahydrobenzo[*d*][1,2,3]selenadiazole group adopts a near to half-chair conformation with puckering parameters of Q = 0.489 (3) Å, θ = 48.9 (4)° and Φ = 218.1 (5)°. The stabilization of crystal packing is mainly governed by intermolecular hydrogen bonding interactions such as C20–H20···O1 (*x* - 1, *y*, *z*) and C22—H22···O1 (Fig. 2). The C—H···π interaction (Fig. 3) is observed between C4—H4A···*Cg* (–x,1 - *y*,1 - *z) [Cg* is the centroid of C16—C21 ring, C···*Cg* distance: 3.584 (3) Å, H···*Cg* 2.63 Å, H-Perp: 2.61 Å], which also contribute to the crystal packing. The crystal packing also exhibits intermolecular π–π stacking interaction between naphthyl units with a centroid-centroid distance of 3.529 (15) Å.

### Experimental

Diastereomerically pure racemic cyclic 1,5-diketones,(*2S*,1'R**)-2-[3-(2-naphthyl)-3-oxo-1-phenylpropyl]cyclohexan-1-one, were obtained via a simple Michael addition of cyclohexanone with substituted chalcones by a known method (Al Arab, 1989; Li *et al.*, 2003; Xu *et al.*, 2009; Qian *et al.*, 2008). A mixture of (*2S*,1'R**)- 2-[3-(2-naphthyl)-3-oxo-1-phenylpropyl]-1-cyclohexanone (1 mmol, 0.36 g) and semicarbazide hydrochloride (1 mmol, 0.11 g) in ethanol (10 ml) was refluxed for 3 h. After completion of the reaction as monitored by TLC, the mixture was poured into ice-cold water (50 ml) and the resulting mono-semicarbazone solid was filtered off. The mono-semicarbazone (1 mmol, 0.41 g) and SeO_2_ (2 mmol, 0.44 g) in THF(10 ml) was refluxed on a water bath for 30 minutes and on completion of the reaction the mixture was filtered to remove selenium powder. The filtrate was concentrated under vacuum, and the residue was subjected to column chromatography using petroleum ether/ethyl acetate mixture (95:5; *v*/*v*) as eluent to afford the racemic mixture of the pure product (Yield: 70%, m.p.: 404-405 K). Dissolving the pure compound in 3:1 mixture of dichloromethane/ethyl acetate and on keeping for slow evaporation of the solvents provides crystals for X-ray analysis.

### Refinement

The hydrogen atoms were placed in calculated positions (C–H = 0.93–0.98 Å) and included in the refinement in riding-model approximation with *U*_iso_(H) = 1.2U_eq_(C).

### Crystal data

**Table:**

C_25_H_22_N_2_OSe	*Z* = 2
*M**_r_* = 445.41	*F*(000) = 456
Triclinic, *P*1	*D*_x_ = 1.450 Mg m^−^^3^
Hall symbol: -P 1	Mo *K*α radiation, λ = 0.71073 Å
*a* = 8.0600 (4) Å	Cell parameters from 3065 reflections
*b* = 10.1215 (5) Å	θ = 2.6–29.3°
*c* = 13.0827 (6) Å	µ = 1.86 mm^−^^1^
α = 81.479 (4)°	*T* = 293 K
β = 76.478 (4)°	Block, blue
γ = 82.087 (4)°	0.4 × 0.3 × 0.2 mm
*V* = 1020.33 (9) Å^3^	

### Data collection

**Table:**

Oxford Diffraction Xcalibur E diffractometer	3594 independent reflections
Radiation source: fine-focus sealed tube	2770 reflections with *I* > 2σ(*I*)
graphite	*R*_int_ = 0.025
Detector resolution: 15.9821 pixels mm^-1^	θ_max_ = 25.0°, θ_min_ = 2.6°
ω scans	*h* = −9→9
Absorption correction: multi-scan (*CrysAlis PRO*; Oxford Diffraction, 2009)	*k* = −12→12
*T*_min_ = 0.658, *T*_max_ = 1.000	*l* = −12→15
6762 measured reflections	

### Refinement

**Table:**

Refinement on *F*^2^	Primary atom site location: structure-invariant direct methods
Least-squares matrix: full	Secondary atom site location: difference Fourier map
*R*[*F*^2^ > 2σ(*F*^2^)] = 0.038	Hydrogen site location: inferred from neighbouring sites
*wR*(*F*^2^) = 0.096	H-atom parameters constrained
*S* = 1.01	*w* = 1/[σ^2^(*F*_o_^2^) + (0.050*P*)^2^] where *P* = (*F*_o_^2^ + 2*F*_c_^2^)/3
3594 reflections	(Δ/σ)_max_ = 0.001
262 parameters	Δρ_max_ = 0.29 e Å^−^^3^
0 restraints	Δρ_min_ = −0.41 e Å^−^^3^

### Special details

**Table:**

Geometry. All e.s.d.'s (except the e.s.d. in the dihedral angle between two l.s. planes) are estimated using the full covariance matrix. The cell e.s.d.'s are taken into account individually in the estimation of e.s.d.'s in distances, angles and torsion angles; correlations between e.s.d.'s in cell parameters are only used when they are defined by crystal symmetry. An approximate (isotropic) treatment of cell e.s.d.'s is used for estimating e.s.d.'s involving l.s. planes.
Refinement. Refinement of *F*^2^ against ALL reflections. The weighted *R*-factor *wR* and goodness of fit *S* are based on *F*^2^, conventional *R*-factors *R* are based on *F*, with *F* set to zero for negative *F*^2^. The threshold expression of *F*^2^ > σ(*F*^2^) is used only for calculating *R*-factors(gt) *etc*. and is not relevant to the choice of reflections for refinement. *R*-factors based on *F*^2^ are statistically about twice as large as those based on *F*, and *R*- factors based on ALL data will be even larger.

### Fractional atomic coordinates and isotropic or equivalent isotropic displacement parameters (Å^2^)

**Table:**

	*x*	*y*	*z*	*U*_iso_*/*U*_eq_	
Se1	0.62678 (4)	0.82915 (3)	0.61536 (3)	0.06072 (16)	
O1	0.3614 (3)	0.5213 (2)	0.20388 (17)	0.0633 (6)	
N1	0.5844 (3)	0.6746 (2)	0.48187 (19)	0.0476 (6)	
N2	0.7071 (3)	0.7047 (3)	0.5160 (2)	0.0587 (7)	
C1	0.4227 (3)	0.7366 (2)	0.5202 (2)	0.0344 (6)	
C2	0.4117 (4)	0.8232 (3)	0.5918 (2)	0.0395 (7)	
C3	0.2505 (4)	0.8991 (3)	0.6443 (2)	0.0485 (7)	
H3A	0.2499	0.9934	0.6163	0.058*	
H3B	0.2451	0.8916	0.7197	0.058*	
C4	0.0939 (4)	0.8443 (3)	0.62557 (19)	0.0422 (7)	
H4A	0.0714	0.7629	0.6736	0.051*	
H4B	−0.0057	0.9098	0.6406	0.051*	
C5	0.1215 (3)	0.8137 (3)	0.5122 (2)	0.0398 (6)	
H5A	0.1464	0.8947	0.4642	0.048*	
H5B	0.0169	0.7858	0.5016	0.048*	
C6	0.2686 (3)	0.7034 (3)	0.48589 (19)	0.0339 (6)	
H6	0.2328	0.6210	0.5303	0.041*	
C7	0.3076 (3)	0.6729 (2)	0.36983 (19)	0.0331 (6)	
H7	0.4113	0.6089	0.3604	0.040*	
C8	0.3471 (3)	0.7946 (3)	0.28883 (18)	0.0334 (6)	
C9	0.5157 (4)	0.8233 (3)	0.2486 (2)	0.0480 (7)	
H9	0.6049	0.7671	0.2711	0.058*	
C10	0.5519 (5)	0.9349 (3)	0.1752 (2)	0.0606 (9)	
H10	0.6651	0.9530	0.1493	0.073*	
C11	0.4227 (5)	1.0187 (3)	0.1405 (2)	0.0601 (9)	
H11	0.4479	1.0933	0.0912	0.072*	
C12	0.2555 (5)	0.9919 (3)	0.1790 (2)	0.0533 (8)	
H12	0.1669	1.0484	0.1560	0.064*	
C13	0.2194 (4)	0.8812 (3)	0.2518 (2)	0.0415 (7)	
H13	0.1057	0.8639	0.2770	0.050*	
C14	0.1634 (3)	0.6015 (3)	0.3511 (2)	0.0376 (6)	
H14A	0.1320	0.5334	0.4105	0.045*	
H14B	0.0635	0.6662	0.3483	0.045*	
C15	0.2124 (4)	0.5363 (3)	0.2507 (2)	0.0375 (6)	
C16	0.0773 (3)	0.4844 (2)	0.2104 (2)	0.0334 (6)	
C17	0.1294 (3)	0.4041 (2)	0.13129 (19)	0.0358 (6)	
H17	0.2463	0.3836	0.1048	0.043*	
C18	0.0114 (3)	0.3514 (2)	0.08876 (19)	0.0340 (6)	
C19	−0.1658 (3)	0.3833 (3)	0.12857 (19)	0.0352 (6)	
C20	−0.2175 (3)	0.4677 (3)	0.2103 (2)	0.0410 (7)	
H20	−0.3339	0.4906	0.2366	0.049*	
C21	−0.1000 (3)	0.5157 (3)	0.2508 (2)	0.0367 (6)	
H21	−0.1367	0.5694	0.3055	0.044*	
C22	−0.2856 (4)	0.3321 (3)	0.0848 (2)	0.0485 (8)	
H22	−0.4026	0.3538	0.1098	0.058*	
C23	−0.2302 (4)	0.2516 (3)	0.0068 (2)	0.0550 (9)	
H23	−0.3095	0.2182	−0.0214	0.066*	
C24	−0.0545 (4)	0.2186 (3)	−0.0316 (2)	0.0532 (8)	
H24	−0.0184	0.1623	−0.0845	0.064*	
C25	0.0639 (4)	0.2670 (3)	0.0068 (2)	0.0441 (7)	
H25	0.1802	0.2450	−0.0205	0.053*	

### Atomic displacement parameters (Å^2^)

**Table:**

	*U*^11^	*U*^22^	*U*^33^	*U*^12^	*U*^13^	*U*^23^
Se1	0.0506 (2)	0.0778 (3)	0.0659 (2)	−0.01457 (18)	−0.02624 (18)	−0.01871 (18)
O1	0.0281 (12)	0.0930 (17)	0.0786 (15)	−0.0050 (11)	−0.0061 (11)	−0.0520 (13)
N1	0.0352 (14)	0.0520 (15)	0.0575 (15)	0.0042 (12)	−0.0141 (12)	−0.0141 (12)
N2	0.0365 (15)	0.0715 (18)	0.0714 (18)	0.0003 (13)	−0.0198 (14)	−0.0115 (14)
C1	0.0340 (16)	0.0341 (14)	0.0349 (14)	−0.0014 (12)	−0.0092 (12)	−0.0025 (12)
C2	0.0424 (17)	0.0410 (15)	0.0380 (15)	−0.0054 (13)	−0.0138 (13)	−0.0049 (12)
C3	0.056 (2)	0.0458 (17)	0.0457 (17)	−0.0006 (15)	−0.0107 (15)	−0.0160 (14)
C4	0.0400 (17)	0.0445 (16)	0.0394 (16)	0.0021 (14)	−0.0051 (14)	−0.0081 (13)
C5	0.0312 (15)	0.0499 (16)	0.0382 (15)	0.0044 (13)	−0.0075 (13)	−0.0130 (13)
C6	0.0320 (15)	0.0353 (14)	0.0352 (14)	−0.0048 (12)	−0.0076 (12)	−0.0053 (11)
C7	0.0261 (14)	0.0340 (14)	0.0404 (15)	0.0014 (11)	−0.0080 (12)	−0.0119 (12)
C8	0.0378 (16)	0.0356 (14)	0.0307 (13)	−0.0041 (12)	−0.0073 (12)	−0.0163 (11)
C9	0.0389 (17)	0.0519 (18)	0.0536 (18)	−0.0098 (14)	−0.0043 (15)	−0.0126 (15)
C10	0.061 (2)	0.065 (2)	0.0523 (19)	−0.028 (2)	0.0107 (18)	−0.0137 (17)
C11	0.088 (3)	0.051 (2)	0.0368 (17)	−0.017 (2)	0.0017 (19)	−0.0073 (15)
C12	0.078 (2)	0.0471 (18)	0.0356 (16)	−0.0009 (17)	−0.0170 (17)	−0.0052 (14)
C13	0.0439 (17)	0.0452 (16)	0.0364 (15)	−0.0020 (14)	−0.0086 (13)	−0.0110 (13)
C14	0.0313 (15)	0.0372 (15)	0.0463 (16)	−0.0039 (12)	−0.0066 (13)	−0.0138 (12)
C15	0.0301 (16)	0.0372 (15)	0.0474 (16)	−0.0004 (12)	−0.0108 (14)	−0.0114 (12)
C16	0.0299 (15)	0.0296 (13)	0.0413 (15)	−0.0013 (11)	−0.0090 (12)	−0.0064 (12)
C17	0.0289 (14)	0.0379 (15)	0.0413 (15)	0.0016 (12)	−0.0093 (12)	−0.0089 (12)
C18	0.0374 (16)	0.0317 (14)	0.0331 (14)	−0.0025 (12)	−0.0103 (12)	−0.0010 (11)
C19	0.0359 (16)	0.0373 (14)	0.0337 (14)	−0.0076 (12)	−0.0097 (12)	−0.0018 (12)
C20	0.0276 (15)	0.0471 (16)	0.0471 (16)	−0.0019 (13)	−0.0038 (13)	−0.0106 (13)
C21	0.0330 (15)	0.0361 (14)	0.0428 (15)	−0.0019 (12)	−0.0068 (13)	−0.0141 (12)
C22	0.0384 (17)	0.066 (2)	0.0453 (17)	−0.0154 (15)	−0.0092 (14)	−0.0096 (15)
C23	0.057 (2)	0.071 (2)	0.0461 (17)	−0.0244 (18)	−0.0162 (16)	−0.0134 (16)
C24	0.065 (2)	0.061 (2)	0.0414 (16)	−0.0106 (17)	−0.0159 (16)	−0.0187 (15)
C25	0.0448 (18)	0.0493 (17)	0.0398 (16)	−0.0006 (14)	−0.0099 (14)	−0.0135 (13)

### Geometric parameters (Å, °)

**Table:**

Se1—C2	1.840 (3)	C20—C21	1.362 (3)
Se1—N2	1.888 (3)	C20—C19	1.417 (4)
O1—C15	1.214 (3)	C20—H20	0.9300
C1—C2	1.355 (4)	C5—H5A	0.9700
C1—N1	1.381 (3)	C5—H5B	0.9700
C1—C6	1.509 (3)	C13—C12	1.375 (4)
N1—N2	1.264 (3)	C13—H13	0.9300
C14—C15	1.503 (3)	C21—H21	0.9300
C14—C7	1.533 (3)	C18—C25	1.421 (4)
C14—H14A	0.9700	C18—C19	1.411 (4)
C14—H14B	0.9700	C25—C24	1.353 (4)
C15—C16	1.499 (3)	C25—H25	0.9300
C17—C16	1.365 (3)	C9—C10	1.385 (4)
C17—C18	1.404 (3)	C9—H9	0.9300
C17—H17	0.9300	C19—C22	1.419 (3)
C7—C8	1.518 (3)	C22—C23	1.356 (4)
C7—C6	1.546 (3)	C22—H22	0.9300
C7—H7	0.9800	C10—C11	1.369 (5)
C6—C5	1.526 (4)	C10—H10	0.9300
C6—H6	0.9800	C12—C11	1.373 (5)
C8—C13	1.386 (4)	C12—H12	0.9300
C8—C9	1.392 (4)	C3—H3A	0.9700
C16—C21	1.412 (4)	C3—H3B	0.9700
C2—C3	1.488 (4)	C23—C24	1.398 (5)
C4—C5	1.520 (3)	C23—H23	0.9300
C4—C3	1.529 (3)	C24—H24	0.9300
C4—H4A	0.9700	C11—H11	0.9300
C4—H4B	0.9700		
			
C2—Se1—N2	86.70 (11)	C4—C5—C6	112.0 (2)
C2—C1—N1	116.2 (2)	C4—C5—H5A	109.2
C2—C1—C6	123.1 (2)	C6—C5—H5A	109.2
N1—C1—C6	120.6 (2)	C4—C5—H5B	109.2
N2—N1—C1	117.3 (2)	C6—C5—H5B	109.2
C15—C14—C7	113.2 (2)	H5A—C5—H5B	107.9
C15—C14—H14A	108.9	C12—C13—C8	122.1 (3)
C7—C14—H14A	108.9	C12—C13—H13	119.0
C15—C14—H14B	108.9	C8—C13—H13	119.0
C7—C14—H14B	108.9	C20—C21—C16	120.3 (2)
H14A—C14—H14B	107.8	C20—C21—H21	119.8
O1—C15—C16	119.7 (2)	C16—C21—H21	119.8
O1—C15—C14	120.5 (2)	C17—C18—C25	122.4 (2)
C16—C15—C14	119.8 (2)	C17—C18—C19	119.0 (2)
C16—C17—C18	121.8 (2)	C25—C18—C19	118.6 (2)
C16—C17—H17	119.1	C24—C25—C18	120.3 (3)
C18—C17—H17	119.1	C24—C25—H25	119.9
C8—C7—C14	112.5 (2)	C18—C25—H25	119.9
C8—C7—C6	113.86 (19)	C10—C9—C8	120.6 (3)
C14—C7—C6	110.17 (19)	C10—C9—H9	119.7
C8—C7—H7	106.6	C8—C9—H9	119.7
C14—C7—H7	106.6	C22—C19—C20	122.3 (3)
C6—C7—H7	106.6	C22—C19—C18	119.3 (2)
N1—N2—Se1	110.7 (2)	C20—C19—C18	118.4 (2)
C1—C6—C5	108.8 (2)	C23—C22—C19	120.3 (3)
C1—C6—C7	113.7 (2)	C23—C22—H22	119.9
C5—C6—C7	114.0 (2)	C19—C22—H22	119.9
C1—C6—H6	106.6	C11—C10—C9	120.7 (3)
C5—C6—H6	106.6	C11—C10—H10	119.6
C7—C6—H6	106.6	C9—C10—H10	119.7
C13—C8—C9	117.2 (3)	C11—C12—C13	119.8 (3)
C13—C8—C7	122.2 (2)	C11—C12—H12	120.1
C9—C8—C7	120.6 (3)	C13—C12—H12	120.1
C17—C16—C21	119.2 (2)	C2—C3—C4	110.6 (2)
C17—C16—C15	118.1 (2)	C2—C3—H3A	109.5
C21—C16—C15	122.7 (2)	C4—C3—H3A	109.5
C1—C2—C3	125.2 (2)	C2—C3—H3B	109.5
C1—C2—Se1	109.2 (2)	C4—C3—H3B	109.5
C3—C2—Se1	125.65 (19)	H3A—C3—H3B	108.1
C5—C4—C3	111.4 (2)	C22—C23—C24	120.4 (3)
C5—C4—H4A	109.3	C22—C23—H23	119.8
C3—C4—H4A	109.3	C24—C23—H23	119.8
C5—C4—H4B	109.3	C25—C24—C23	121.2 (3)
C3—C4—H4B	109.3	C25—C24—H24	119.4
H4A—C4—H4B	108.0	C23—C24—H24	119.4
C21—C20—C19	121.2 (3)	C10—C11—C12	119.6 (3)
C21—C20—H20	119.4	C10—C11—H11	120.2
C19—C20—H20	119.4	C12—C11—H11	120.2
			
C2—C1—N1—N2	−0.6 (4)	C3—C4—C5—C6	63.3 (3)
C6—C1—N1—N2	−178.3 (2)	C1—C6—C5—C4	−49.3 (3)
C7—C14—C15—O1	13.6 (4)	C7—C6—C5—C4	−177.3 (2)
C7—C14—C15—C16	−168.8 (2)	C9—C8—C13—C12	−0.5 (4)
C15—C14—C7—C8	67.1 (3)	C7—C8—C13—C12	179.5 (2)
C15—C14—C7—C6	−164.7 (2)	C19—C20—C21—C16	1.3 (4)
C1—N1—N2—Se1	0.9 (3)	C17—C16—C21—C20	−0.9 (4)
C2—Se1—N2—N1	−0.8 (2)	C15—C16—C21—C20	179.0 (2)
C2—C1—C6—C5	19.6 (3)	C16—C17—C18—C25	−179.6 (2)
N1—C1—C6—C5	−162.9 (2)	C16—C17—C18—C19	0.4 (4)
C2—C1—C6—C7	147.8 (2)	C17—C18—C25—C24	179.9 (3)
N1—C1—C6—C7	−34.7 (3)	C19—C18—C25—C24	−0.1 (4)
C8—C7—C6—C1	−69.0 (3)	C13—C8—C9—C10	0.5 (4)
C14—C7—C6—C1	163.5 (2)	C7—C8—C9—C10	−179.5 (2)
C8—C7—C6—C5	56.5 (3)	C21—C20—C19—C22	−179.9 (2)
C14—C7—C6—C5	−71.0 (3)	C21—C20—C19—C18	−0.9 (4)
C14—C7—C8—C13	39.2 (3)	C17—C18—C19—C22	179.1 (2)
C6—C7—C8—C13	−87.1 (3)	C25—C18—C19—C22	−0.9 (4)
C14—C7—C8—C9	−140.8 (2)	C17—C18—C19—C20	0.0 (3)
C6—C7—C8—C9	92.9 (3)	C25—C18—C19—C20	−179.9 (2)
C18—C17—C16—C21	0.0 (4)	C20—C19—C22—C23	−179.9 (3)
C18—C17—C16—C15	−179.9 (2)	C18—C19—C22—C23	1.0 (4)
O1—C15—C16—C17	9.8 (4)	C8—C9—C10—C11	−0.3 (4)
C14—C15—C16—C17	−167.9 (2)	C8—C13—C12—C11	0.3 (4)
O1—C15—C16—C21	−170.1 (3)	C1—C2—C3—C4	12.4 (4)
C14—C15—C16—C21	12.3 (4)	Se1—C2—C3—C4	−166.41 (18)
N1—C1—C2—C3	−179.1 (2)	C5—C4—C3—C2	−41.7 (3)
C6—C1—C2—C3	−1.5 (4)	C19—C22—C23—C24	−0.2 (4)
N1—C1—C2—Se1	−0.1 (3)	C18—C25—C24—C23	1.0 (4)
C6—C1—C2—Se1	177.50 (18)	C22—C23—C24—C25	−0.8 (5)
N2—Se1—C2—C1	0.47 (19)	C9—C10—C11—C12	0.1 (5)
N2—Se1—C2—C3	179.4 (2)	C13—C12—C11—C10	−0.1 (4)

### Hydrogen-bond geometry (Å, °)

**Table:**

Cg is the centroid of the C16–C21 ring.

**Table:**

*D*—H···*A*	*D*—H	H···*A*	*D*···*A*	*D*—H···*A*
C20—H20···O1^i^	0.93	2.56	3.381 (3)	147
C22—H22···O1^i^	0.93	2.58	3.392 (4)	146
C4—H4A···Cg^ii^	0.97	2.63	3.584 (3)	167

Symmetry codes: (i) *x*−1, *y*, *z*; (ii) −*x*, −*y*+1, −*z*+1.

## References

[bb1] Al Arab, M. M. (1989). *J. Chem. Soc. Pak.* **11**, 321–326.

[bb2] Cremer, D. & Pople, J. A. (1975). *J. Am. Chem. Soc.* **97**, 1354–1358.

[bb3] El-Bahaie, S., Assy, M. G. & Hassanien, M. M. (1990). *Pharmazie*, **45**, 791–793. 2089395

[bb4] El-Kashef, H. S., E-Bayoumy, B. & Aly, T. I. (1986). *Egypt. J. Pharm. Sci.* **27**, 27–30.

[bb5] Farrugia, L. J. (1997). *J. Appl. Cryst.* **30**, 565.

[bb6] Kuroda, K., Uchikurohane, T., Tajima, S. & Tsubata, K. (2001). US Patent 6 166 054.

[bb7] Li, J. T., Chen, G.-F., Xu, W.-Z. & Li, T.-S. (2003). *Ultrason. Sonochem.* **10**, 115–118. 10.1016/s1350-4177(02)00134-7 12551772

[bb8] Oxford Diffraction (2009). *CrysAlis PRO* Oxford Diffraction Ltd, Yarnton, England.

[bb9] Plano, D., Moreno, E., Font, M., Encío, I., Palop, J. A. & Sanmartín, C. (2010). *Arch. Pharm.* **343**, 680–691. 10.1002/ardp.201000014 21110339

[bb10] Qian, Y., Xiao, S., Liu, L. & Wang, Y. (2008). *Tetrahedron Asymmetry*, **19**, 1515–1518.

[bb11] Sheldrick, G. M. (2008). *Acta Cryst.* A**64**, 112–122. 10.1107/S0108767307043930 18156677

[bb12] Spek, A. L. (2009). *Acta Cryst.* D**65**, 148–155. 10.1107/S090744490804362X PMC2631630 19171970

[bb13] Xu, D.-Z., Shi, S., Liu, Y. & Wang, Y. (2009). *Tetrahedron*, **65**, 9344–9349.

